# European and African-specific plasma protein-QTL and metabolite-QTL analyses identify ancestry-specific T2D effector proteins and metabolites

**DOI:** 10.21203/rs.3.rs-3617016/v1

**Published:** 2024-07-22

**Authors:** Carlos Cruchaga, Chengran Yang, Priyanka Gorijala, Jigyasha Timsina, Lihua Wang, Menghan Liu, Ciyang Wang, William Brock, Yueyao Wang, Yun Ju Sung

**Affiliations:** Washington University; Washington University in St. Louis; Washington University School of Medicine; Department of Psychiatry, Washington University School of Medicine, St. Louis, MO, USA; Washington University School of Medicine; Washington University School of Medicine, St. Louis, MO, USA; Washington University School of Medicine; Washington University School of Medicine, St. Louis, MO, USA; Washington University School of Medicine, St. Louis, MO, USA; Washington University Medical School

## Abstract

Initially focused on the European population, multiple genome-wide association studies (GWAS) of complex diseases, such as type-2 diabetes (T2D), have now extended to other populations. However, to date, few ancestry-matched omics datasets have been generated or further integrated with the disease GWAS to nominate the key genes and/or molecular traits underlying the disease risk loci. In this study, we generated and integrated plasma proteomics and metabolomics with array-based genotype datasets of European (EUR) and African (AFR) ancestries to identify ancestry-specific muti-omics quantitative trait loci (QTLs). We further applied these QTLs to ancestry-stratified T2D risk to pinpoint key proteins and metabolites underlying the disease-associated genetic loci. We nominated five proteins and four metabolites in the European group and one protein and one metabolite in the African group to be part of the molecular pathways of T2D risk in an ancestry-stratified manner. Our study demonstrates the integration of genetic and omic studies of different ancestries can be used to identify distinct effector molecular traits underlying the same disease across diverse populations. Specifically, in the AFR proteomic findings on T2D, we prioritized the protein QSOX2; while in the AFR metabolomic findings, we pinpointed the metabolite GlcNAc sulfate conjugate of C21H34O2 steroid. Neither of these findings overlapped with the corresponding EUR results.

## Introduction

Human genetics studies have mainly focused on participants of European ancestry^1^. However, there has been a recent increase in studies that include multiple ancestral backgrounds^2–4^. With these efforts, human geneticists now have published numerous studies on complex diseases that encompass multiple populations or specifically target non-European populations. For example, two of the largest studies on type-2 diabetes (T2D) included participants of five different ancestries^5,6^ - Europeans, Africans, Hispanics, East Asians, and South Asians. However, there has been a lack of utilization of ancestry-matched deep molecular phenotyping datasets in post-GWAS analyses, namely colocalization^7,8^, Mendelian Randomization^9,10^ or trait-imputation (such as Transcriptome-wide association study (TWAS) for RNA expression^11,12^). These approaches are pivotal to prioritizing variants to genes or further to pathways.

Recent publications have included single-layer omics genetic studies that incorporate participants from multiple ancestries rather than just a single ancestry. In 2022, two proteomic studies were published: (i) Zhang et al.,^13^ investigated plasma proteomic data from participants of European and African ancestries within the Atherosclerosis Risk in Communities (ARIC) cohort; (ii) Schubert et al.,^14^ examined plasma proteomic data from African American, East Asian, European, and Hispanics participants within the Multi-Ethnic Study of Atherosclerosis (MESA) cohort. Both studies applied the TWAS framework to study the cis-only protein effects on diseases. Consequently, they coined the term PWAS specifically for studying the proteome. Additionally, a more recent study, published in 2023 with the largest-to-date sample size by Sun et al., used the UK Biobank Pharma Proteomics Project (UKB-PPP) cohort^15^ and generated genome-wide multi-ancestry protein-QTL (pQTL) datasets.

Meanwhile, single ancestry multi-omic genetic studies have also been reported before^16,17^. Battle et al.,^16^ performed the three layers of cis-QTL mapping from RNA expression, translated RNA to protein levels in the African lymphoblastoid cell lines. Brown et al.,^17^ mapped a different set of three modalities of QTLs from transcriptomic (cis and trans for 16,206 genes, cis only for 170,198 exons and 64,546 splicing phenotypes), proteomic (cis and trans for 373 proteins) and metabolomic (genome-wide regions for 116 metabolites) data from blood or plasma samples from European individuals. However, no studies have been performed integrating genetics of complex traits with multi-omics in an ancestry-matched manner.

**Figure 1:**
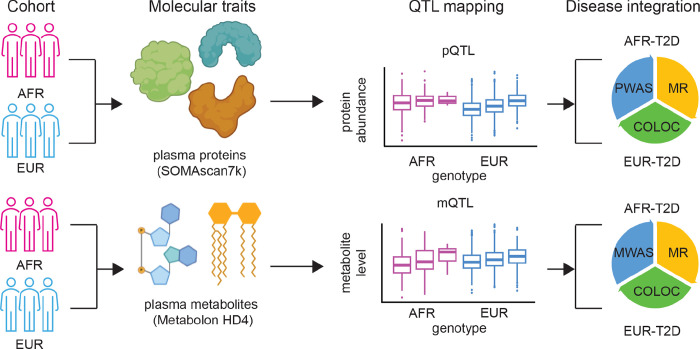
Schematics of the study design on genetic architecture of the plasma proteome and metabolome in participants with African and European ancestry. Top: The plasma proteins in participants of African and European ancestries were profiled together with the SOMAscan 7k platform. Integrating the abundance of each protein with the array-based genotype data, we identified pQTLs in both ancestries. We further used these pQTLs to prioritize proteins in the ancestry-matched T2D risk via three methods, PWAS, MR, and COLOC. Bottom: The plasma metabolites in participants of African and European ancestries were profiled together with the Metabolon HD4 platform. Integrating the level of each metabolite with the array-based genotype data, we identified mQTLs in both ancestries. We further used these mQTLs to prioritize metabolites in the ancestry-matched T2D risk via three methods, PWAS, MR, and COLOC.

Here, we aimed to first identify the ancestry-specific genetic associations in both the plasma proteome and metabolome (Fig. 1). Next, we applied them to pinpoint key proteins/metabolites underlying the risk loci of ancestry-matched T2D. Our study used plasma omics to study T2D for two reasons: 1) T2D GWAS have the largest sample sizes in both European (EUR) and African (AFR) ancestries. This provides more genome-wide significant loci to consider when studying this disease; 2) plasma analytes are more relevant to metabolic disorders, such as T2D, compared to diseases that are enriched in certain tissue types, such as the brain for neurological disorders. In our study, we also considered trans associations in all our molecular trait QTL mapping sets. By incorporating trans associations, we anticipate uncovering more findings compared to those previous cis-centric studies.

## Results

### Genetic architecture of the plasma proteome in participants of African and European ancestry

To build genetic maps of the plasma proteome and metabolome, we performed pQTL and metabolite-QTL (mQTL) analyses separately (Fig. 1; Figure S3A-F, Table S2–7). To obtain the ancestry stratified maps, we further split the input data into African and European sub-cohorts. In summary, we utilized an aptamer-based assay (SOMAscan 7k platform^18^) to measure the multi-ancestry proteomics data and a mass-spectrometry assay (Metabolon HD4 platform^19^) for the metabolomics data of the same cohort. Following quality control procedures for the omics data and integration with array-based post-imputation genotype data, we constructed four maps: i) AFR pQTL (414 participants and 6,907 proteins), ii) EUR pQTL (2,338 participants and 6,907 proteins), iii) AFR mQTL (417 participants and 1,413 metabolites), and iv) EUR mQTL (2,392 participants and 1,483 metabolites). To determine the study-wide significant QTLs, we derived it from genome-wide significance after further accounting for the number of independent features within each separate map (see Methods for more details).

**Figure 2:**
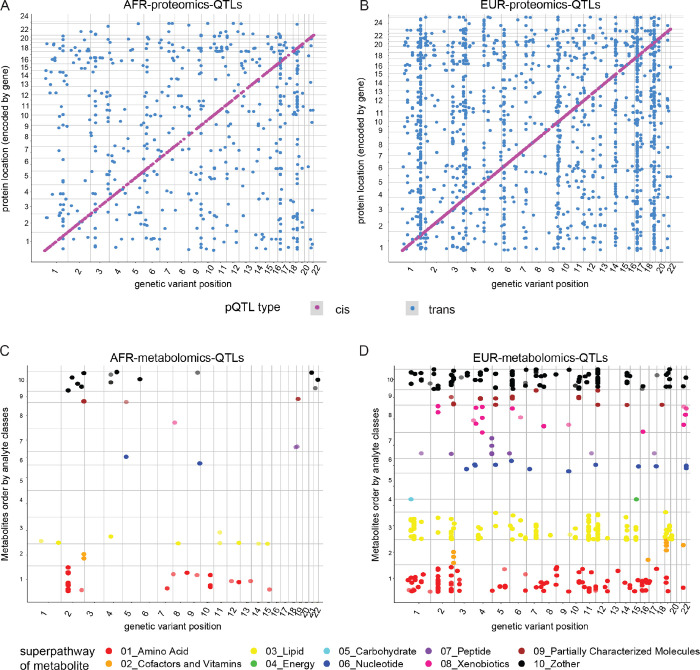
Four genetic maps of the plasma proteome and metabolome in participants with African and European ancestry. **A)** Map of 954 AFR pQTLs. The X-axis is the genetic variant positions on the genome (hg38); the Y-axis is the protein location (encoded by its corresponding gene, hg38). The color code is magenta as cis-pQTLs and blue as trans-pQTLs. **B)**Map of 2,848 EUR pQTLs. Same X, Y, and color code as panel-A. **C)** Map of 65 AFR mQTLs. The X-axis is the genetic variant positions on the genome (hg38); the Y-axis is the metabolite order (based on the super pathway). The color code is red as 01_Amino Acid; orange as 02_Cofactors and Vitamins; yellow as 03_Lipid; limegreen as 04_Energy; cyan as 05_Carbohydrate; blue as 06_Nuleotide; purple as 07_Peptide; deepplink1 as 08_Xenobiotics; brown as 09_Partially Characterized Molecules; black as 10_other categories. **D)** Map of 490 EUR mQTLs. Same X, Y, and color code as panel-C.

To identify genetic variants associated with the plasma proteome in individuals of African ancestry, we conducted pQTL mapping on African ancestry participants (Fig. 2A). Of 6,907 proteins that passed QC, we identified 881 proteins with 954 study-wide significant pQTLs (Table S11). Among these findings, 420 pQTLs were classified as *cis*, while 534 were *trans*-pQTLs. Consistent with previous studies^20,15,21^, we observed that the absolute effect size was negatively correlated with the minor allele frequency (Figure S4A). After assigning each pQTL to its corresponding linkage disequilibrium (LD) block^22^, we identified a total of 508 unique genetic loci, including 84 pleiotropic regions. Notably, we found 33 proteins associated with the *APOE* locus (Figure S5A), which ranked second in terms of AFR proteomic-associated pleiotropic regions and genomic hotspots. The other top-five pleiotropic loci included *VTN* (chr17q11.2) with 36 proteins, *ABO* (chr9q34.2) with 24 proteins, *CFH* (chr1q31.3) with 23 proteins, and the *MHC* region with 22 proteins. Next, we performed a stratified analysis for individuals of European ancestry. Of the 6,907 proteins, 2,400 proteins showed 2,848 significant pQTLs in this population; 1,282 *cis*-pQTLs and 1,566 *trans*-pQTL (Fig. 2B; Table S12; Figure S4B). Of the top five pleiotropic pQTL loci in EUR (totally 746 regions), the *APOE* locus (chr19q13.32) was associated with 126 proteins (Figure S5B). The other top-five pleiotropic loci included the *VTN* (chr17q11.2) with 182 proteins, *CFH* (chr1q31.3) with 151 proteins, *MHC* region with 86 proteins, and the *ABO* locus (chr9q34.2) with 82 proteins.

To determine how many of the pQTLs have been reported before, we conducted a comparison between our study-wide pQTLs and the three largest to date external studies covering both cis and trans associations while encompassing two genetic ancestries (see Methods). Of these four datasets from three external pQTL studies (Table S13), Ferkingstad et al.,^23^ and Sun et al.,^15^ included participants of EUR ancestry, while Surapaneni et al.,^20^ and Sun et al., ^15^ sampled individuals of AFR ancestry. Overall, out of the 954 AFR pQTLs (Table S14) identified, we found that 561 had been previously reported with a study-wide significant p-value threshold of 5×10^− 11^. Additionally, among the remaining 393 AFR pQTLs, 14 had been reported with a genome-wide threshold, 45 had passed a nominal threshold, and 242 did not show nominal significance in previous studies. Among the pQTLs that were not tested, 82 were due to missing proxy variants, and 10 were due to missing protein data. Considering the largest number of proteins (~ 5k) profiled from a large-scale European cohort in the study by Ferkingstad et al.,^23^, we can replicate the highest number of our findings for the EUR pQTLs, with a p-value below 5×10^− 2^ in this external study. Of the 2,848 EUR pQTLs identified (Table S14), 2,052 had been reported as study-wide significant (p < 5×10^− 11^), 43 were below a genome-wide threshold, 241 were below a nominal threshold, while 395 were above the nominal threshold. This indicates that 86% of the tested pQTLs have supportive evidence from previous studies. Among the untested pQTLs, 81 were due to missing proxy variants and 36 were due to the missing protein data.

### Genetic architecture of the plasma metabolome in participants of African and European ancestry

To detect genetic variants associated with the plasma metabolome in African and European ancestry respectively, we performed mQTL mapping using the same participants from which the proteomic data was generated (Fig. 2C–D). After quality controlling for both genotype and metabolome datasets, we identified 65 significant mQTLs in 34 genetic loci, associated with 60 metabolites (out of a total of 1,413 metabolites tested) in the African-American cohort (Fig. 2C, Table S15). Notably, a significant number of these hits (27 out of 34 loci) were involved in and enriched for the super pathway of amino acids (odds ratio = 2.78, Fisher exact test, p-value = 6.63×10^− 5^) compared to other pathways. In the European-American cohort, we found 490 significant mQTLs in 124 genetic regions associated with 403 metabolites (Fig. 2D, Table S16). The majority of the hits were observed in two super pathways: lipids (198 metabolites, odds ratio = 1.26, Fisher exact test, p-value = 0.019) and amino acids (106 metabolites, odds ratio = 1.52, Fisher exact test, p-value = 0.0015). In line with a previous cross-platform mQTL study^24^, we observed both sets of mQTL followed a trend where the absolute effect size negatively correlated with the minor allele frequency across all variants (Figure S4C-D). Additionally, we identified top-ranked LD blocks associated with metabolites were loci near *ALMS1P1* (chr2p13.1), *UGT1A6* (chr2q37.1), and *PYROXD2* (chr10q24.2) in the African cohort (Figure S5C), and *FADS1/2* (chr11q12.2), *SLCO1B1* (chr12p12.1), *ALMS1P1* (chr2p13.1) in the European-American cohort (Figure S5D). These regions represent metabolite-associated pleiotropic regions and genomic hotspots specific to their respective populations.

To assess the presence of previously reported mQTLs, we examined our study-wide mQTLs using three of the up-to-date external studies with full summary statistics available, which included participants from both genetic ancestries (details see Methods). Among these three external mQTL studies (Table S17), Yin et al.,^25^, and Chen et al.,^26^ focused on individuals of EUR ancestry alone, while Rhee et al.,^27^ included participants of AFR ancestry. Of the 65 AFR mQTLs identified (Table S18), we found that 48 had already been reported after multiple testing corrections with a study-wide significance threshold of 5×10^− 11^. None of these 65 mQTLs were below a genome-wide threshold, one passed a nominal threshold, seven were above the nominal threshold, and nine were not examined as the metabolites were missing. On the other hand, of the 490 EUR mQTLs identified (Table S18), we found 412 passed the study-wide threshold of p-value as 5×10^− 11^, seven passed a genome-wide threshold, ten passed a nominal threshold, while nine did not reach the nominal threshold. Among the mQTLs that were not tested, two were due to the missing proxy variants and 50 were due to the missing metabolite data. These results indicate that approximately 88% of tested mQTL pairs in the African ancestry cohort and 98% of tested mQTL pairs in the European ancestry cohort have supportive evidence from previous studies.

All four QTL datasets can be interactively explored on the PheWeb^28^-based ONTIME browser (https://ontime.wustl.edu/).

### Ancestry-specific pQTLs and mQTLs

To identify ancestry-specific xQTLs (i.e., pQTL and mQTL), we compared results between participants of African and European ancestry. Briefly, ancestry-specific hits were determined based on fold-change criteria and considering both the effect size and standard error (for deriving the Z-normalized effect size), following the methodology used in previous condition-dependent genetic studies^29,30^. The fold-change greater than ten-fold or smaller than 10%-fold was used as the threshold, with log10-based fold change boundaries of +/− 1 to determine context-specific QTLs (see Methods).

**Figure 3:**
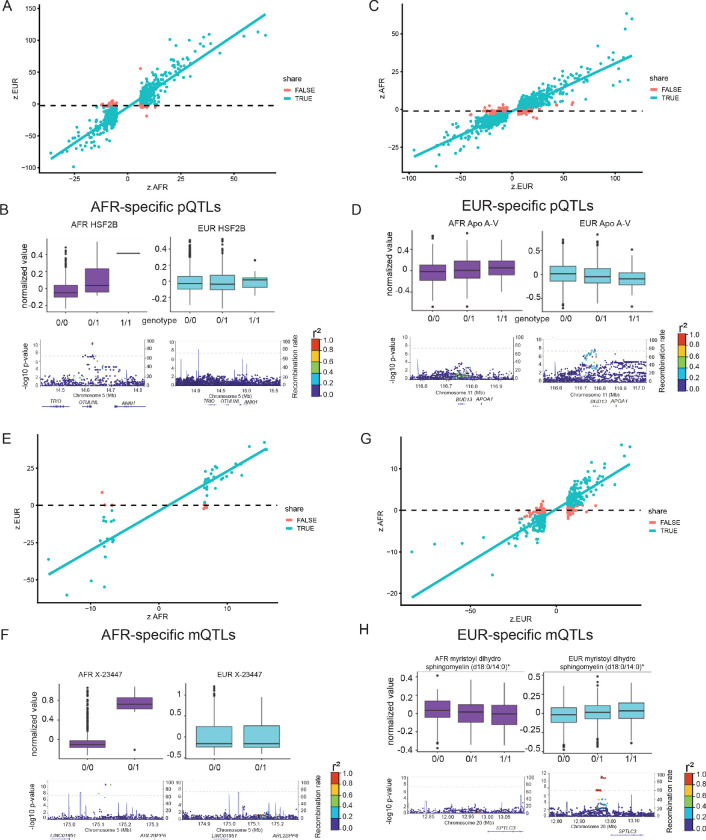
Ancestry-specific pQTL and mQTL hits. **A)** AFR-specific pQTL vs queried EUR. The correlation coefficients of the standardized effect sizes between the two ancestries are 0.923 and −0.147 for the shared (green) and AFR-specific (red) pQTLs. The lines are based on a linear regression model. **B)** One AFR-specific pQTL exampleas HSF2B visualized by boxplots and locus zoom plots. **C)** EUR-specific pQTL vs queried AFR. The correlation coefficients of the standardized effect sizes between the two ancestries are 0.935 and 0.234 for the shared (green) and EUR-specific (red) pQTLs. The lines are based on a linear regression model. **D)** One EUR-specific pQTL example as Apo A-V visualized by boxplots and locus zoom plots. **E)** AFR-specific mQTL vs queried EUR. The correlation coefficients of the standardized effect sizes between the two ancestries are 0.927 and −0.51 for the shared (green) and AFR-specific (red) mQTLs. The lines are based on a linear regression model. **F)** One AFR-specific mQTL example as X-23447 visualized by boxplots and locus zoom plots. **G)**EUR-specific mQTL vs queried AFR. The correlation coefficients of the standardized effect sizes between the two ancestries are 0.927 and −0.51 for the shared (green) and AFR-specific (red) mQTLs. The lines are based on a linear regression model. **H)** One EUR-specific mQTL example as myristoyl dihydrosphingomyelin (d18:0/14:0) visualized by boxplots and locus zoom plots.

In the case of proteomics, of the 954 pQTLs identified in AFR participants, 29.6% were considered AFR-specific pQTLs (Fig. 3A, Table S19). For example, in African-ancestry participants, the protein levels of HSF2B (Heat shock factor 2-binding protein) were positively associated with the genetic variant chr5:14626365:T:C (*Z* = 6.77, *MAF* . *afr* = 0.05), However, in European-ancestry participants, the HSF2B protein levels were similar across all genotypes of the same variant (*Z* = −0.007, *MAF* . *eur* = 0.10), resulting a large fold-change difference (*Z*. *afr*/*Z*. *eurratio* = −949, Fig. 3B). Similarly, among the 2,848 pQTL identified in EUR participants, 24.3% were considered EUR-specific pQTLs (Fig. 3C, Table S20). In the European-ancestry cohort, the protein levels of Apo A-V (Apolipoprotein A-V) were significantly decreased with the minor allele dosages of the variant chr11:116780399:C:T (*Z* = −5.55, *MAF* . *eur* = 0.20) but this association was not observed in African-ancestry participants (*Z* = 2.51, *MAF* . *afr* = 0.26), leading to a fold-change ratio of −0.452 (Fig. 3D).

In the field of metabolomics, we compared mQTL results from participants of the two ancestries. Of the 65 mQTLs identified with AFR participants, 20% were classified as AFR-specific mQTLs (Fig. 3E, Table S21). For instance, in AFR participants, the metabolite abundances of the X-23447 were increased with the minor allele dosage of the genetic variant chr5:175129766:C:T (*Z* = 6.89, *MAF* . *afr* = 0.0096). However, in EUR participants, this metabolite displayed similar levels across all genotypes of the variant (*Z* = −0.0116, *MAF* . *eur* = 0.014), resulting in a substantial fold-change difference (*ratioofZ*. *afr*/*Z*. *eur* = −594, Fig. 3F). On the other hand, among the 490 mQTLs identified with EUR participants, 23.7% were considered EUR-specific mQTLs (Fig. 3G, Table S22). In the EUR cohort, the levels of myristoyl dihydrosphingomyelin (d18:0/14:0) were significantly and positively associated with the minor allele dosages of the variant chr20:12978750:T:C (*Z* = 6.84, *MAF* . *eur* = 0.38), whereas in the AFR group, this association was not observed (*Z* = −1.98, *MAF* . *afr* = 0.41), leading to a fold-change ratio of −0.289 (Fig. 3H).

To further characterize the ancestry-specific QTLs, we categorized the genetic variants into three bins based on minor allele frequency (MAF). The bins were defined as follows: bin-1, MAF ranging from 0 to 0.01; bin-2, MAF ranging from 0.01 to 0.05; and bin-3, MAF ranging from 0.05 to 0.5. The MAF threshold for each variant was determined by considering the minimum MAF value between the two ancestries. We found that the larger the MAF of a genetic variant, the less likely it was to be specific to a particular ancestry (Figure S6A-G). On average, the proportion of ancestry-specific QTLs decreased from 68.5–45% as the MAF bins shifted from bin-1 to bin-2. Moreover, the ancestry-specific QTLs decreased to 10.8% at bin-3. Power analyses (see Methods) indicated that the sentinel xQTLs used in this ancestry specificity section were well-powered given the current sample size. Even though the variants with lower MAF tend to have a lower power with the same effect size, all the ancestry-specific variants showed > 80% power in the other ancestry.

As a complementary strategy, we employed a more flexible Bayesian approach, multivariate adaptive shrinkage (MASH) framework^29^, to calculate the posterior probability and posterior mean for each QTL-trait pair in the two ancestries. The posterior mean fold change also indicated a similar ancestry-sharing proportion ranging from 82.3–96.2% (Figure S7A-D), which supports the previous estimations of ancestry-specific QTLs. These results align with previous studies (Zhang et al.,^13^ for proteomics and Rhee et al.,^27^ for metabolomics datasets). Zhang et al.,^13^ reported 10% EUR-specific and 30% AFR-specific cis-pQTLs, while Rhee et al.,^27^ uncovered 22% ancestry-specific mQTLs. Moreover, our findings extend these previous reports by analyzing different MAF bins, revealing that the ancestry-specific QTLs are more likely to have lower frequencies. This observation could be explained by the fact that functional variants tend to have lower frequencies than non-functional variants, and therefore, these ancestry-specific QTLs may capture those functional variants. Alternatively, participants of African ancestry may have a higher prevalence of rare variants compared to those of European ancestry, which increases the likelihood of finding ancestry-specific associations.

### Integration of proteins and metabolites with the risk of ancestry-matched T2D via PWAS and MWAS

**Figure 4:**
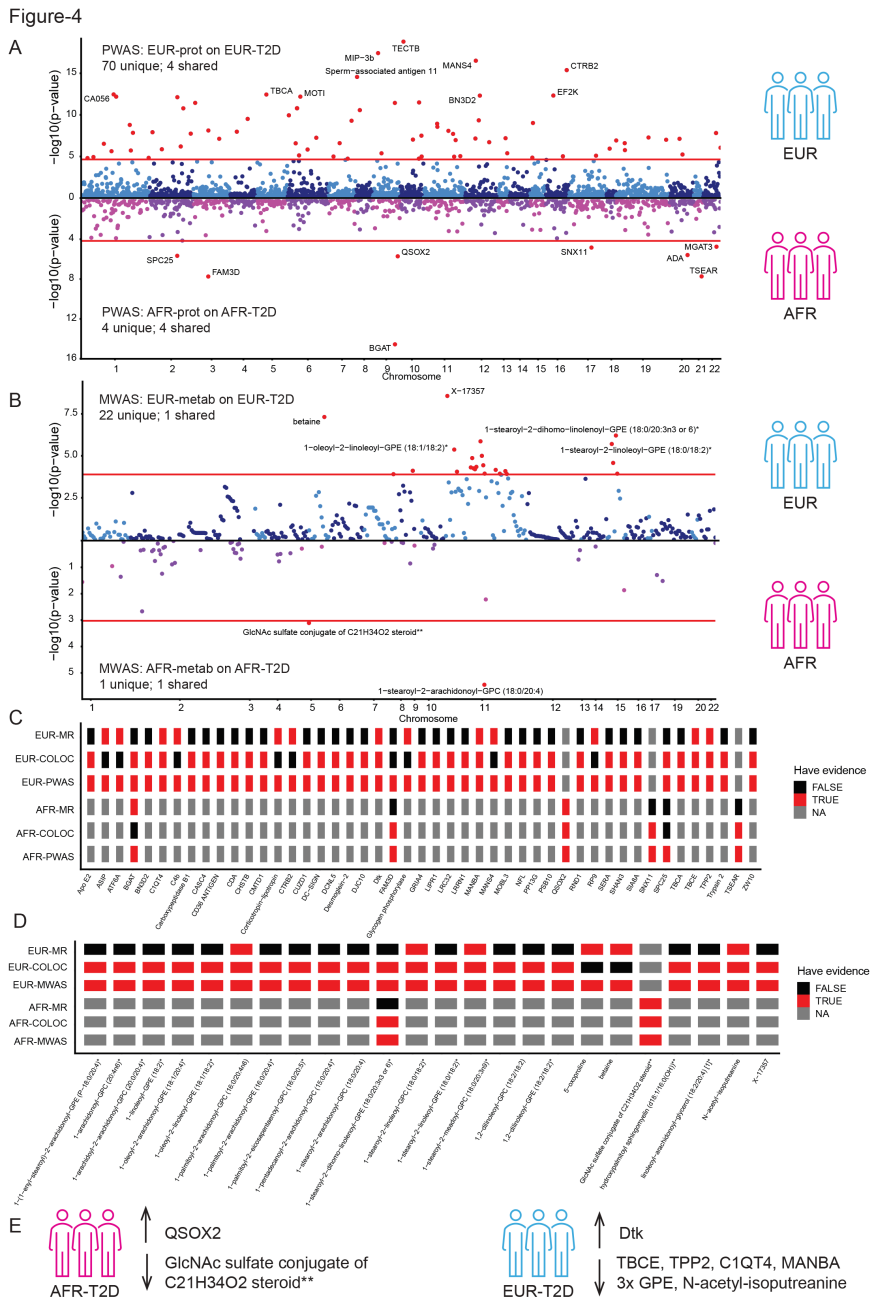
Integration of proteins and metabolites with the ancestry-matched risk of type-2 diabetes. **A)** Miami plot highlighting the top proteins associated with T2D between two ancestries. The red line is based on Bonferroni correction thresholds of the EUR (top) and AFR (bottom) PWAS. **B)**Miami plot highlighting the top metabolites associated with T2D between two ancestries. The red line is based on Bonferroni correction thresholds of the EUR (top) and AFR (bottom) MWAS. **C)** Heatmap of findings per ancestry-matched protein-disease associations integrations with different evidence: i) PWAS (Bonferroni p-value < 0.05); ii) colocalization (PP.H4 > 0.8); iii) MR (FDR < 0.05) within EUR (top) and AFR (bottom) with at least two evidence as TRUE. The color code: red as TRUE, black as FALSE, gray as not available or NA. **D)** Heatmap of findings per ancestry-matched metabolite-disease associations integrations with different evidence: i) MWAS (Bonferroni p-value < 0.05); ii) colocalization (PP.H4 > 0.8); iii) MR (FDR < 0.05) within EUR (top) and AFR (bottom) with at least two evidence as TRUE. The color code: red as TRUE, black as FALSE, gray as not available or NA. **E)** Schematic of the summary of ancestry-stratified proteins or metabolites underlying T2D risk nominated in all three post-GWAS methods.

Finally, to identify proteins associated with ancestry-specific risk of T2D, we first employed the PWAS framework. Specifically, we prioritized proteins that were associated with ancestry-matched T2D risk within each ancestry group, namely EUR- and AFR-stratified analyses (Fig. 4A–B, Figure S8–9, Table S23–24). For the EUR T2D risk analysis, we used the summary statistics from the GWAS published by Mahajan et al.,^5^ that included 80,154 cases and 853,816 controls. On the other hand, to investigate AFR T2D risk, we leveraged the study conducted by Vujkovic et al.,^6^ which enrolled 24,646 cases and 31,446 controls. These two GWAS reported 225 and 23 genome-wide significant hits in EUR and AFR GWAS, respectively.

From the ancestry-stratified PWAS analyses, we found 74 proteins in EUR and eight in AFR associated with T2D after multiple testing corrections (Fig. 4A). In the European ancestry group, the associated proteins included C4b, NFL, BGAT, among others. (Fig. 4A, top). For the African ancestry group, the associated proteins were FAM3D, MGAT3, SPC25, and BGAT (Fig. 4A, bottom). Four proteins (SPC25, BGAT, FAM3D, MGAT3) were found in common in the EUR and AFR-specific analyses.

We applied the same framework to assess the association between metabolite levels and T2D in an ancestry-specific manner (Fig. 4B, Figure S8–9, Table S25–26). We identified 23 EUR and two AFR metabolites given Bonferroni-corrected thresholds (Fig. 4B). Of the 23 significant metabolites from EUR MWAS analysis (Fig. 4B, top), 14 have been studied in differential level analyses concerning T2D, and six of these metabolites were reported to be differentially expressed between T2D cases and controls in a comprehensive external study by Zaghlool et al.,^31^. Furthermore, two noteworthy AFR metabolites from AFR MWAS analysis (Fig. 4B, bottom) that exhibited significance were GlcNAc sulfate conjugate of C21H34O2 steroid and 1-stearoyl-2-arachidonoyl-GPC. Neither of them had been reported previously in the differentially expression study^31^. One metabolite, 1-stearoyl-2-arachidonoyl-GPC (18:0/20:4), was in common between EUR and AFR groups.

### Integration of proteins and metabolites with the risk of ancestry-matched T2D via genetic colocalization and Mendelian randomization

To minimize the possibility of false-positive findings in XWAS (including PWAS and MWAS), we implemented two supplementary analyses: genetic colocalization (Fig. 4C–D, Table S27–30) and Mendelian randomization (Fig. 4C–D, Table S31–34) between the molecular traits and T2D risk).

In the EUR-stratified analyses (Figure S10A-B), there were 36 proteins and 21 metabolites significant for the XWAS and colocalization analyses; and 13 proteins and six metabolites significant for the XWAS and MR. In the AFR-specific analyses (Figure S10C-D), four proteins and two metabolites were significant on the XWAS and colocalization analyses. Moreover, two proteins were significant in both PWAS and MR.

To emphasize the proteins and metabolites with the highest confidence in terms of their association with the T2D risk, we only highlighted those that are significant after multiple test correction in all three analyses: XWAS, colocalization, and MR. Consequently, we pinpointed five proteins and four metabolites in the EUR-specific (Figure S11A-E, Figure S12A-D) and one protein and one metabolites the AFR-specific analyses (Figure S11F; Figure S12E; Table S35–38). No proteins were in common between the EUR- and AFR-specific analyses (Fig. 4E).

In the AFR-specific analyses (PWAS, colocalization, MR), we nominated a protein called QSOX2 (Fig. 4E, Figure S11F). This protein is regulated by a *trans*-pQTL under the known risk gene, *ABO*^6^ (Table S35). It is worth noting that the variant chr9:133252214:G:A used as an instrumental variable was not in high LD (r^2^ = 4×10^− 4^) with the closest pleiotropic variant chr9:133254260:G:A, and hence it was included in the analysis). QSOX2 showed a positive association with T2D risk (PWAS.Z = 4.763 and MR.beta = 0.246). Additionally, the posterior probability of genetic colocalization between QSOX2 and the disease was high (PP.H4 = 0.977). Although QSOX2 has been previously annotated in the process of protein folding^32^, it has not been published as a known T2D effector.

Of the five EUR proteins associated with EUR T2D^5^ (Fig. 4E, Table S36, Figure S11A-E), Dtk, C1QT4, and MANBA were encoded by genes that were already known to be implicated in T2D. In this study, these proteins were nominated based on the PWAS, colocalization and MR results. These results were driven by cis-pQTLs to these proteins. These loci were reported as being genome-wide significant and those genes were nominated based on based on variant annotation, genetic colocalization with eQTLs, pcHi-C links, and TWAS significance^5,6^., Dtk has been reported to be involved in the positive regulation of kinase activity^32^; C1QT4 plays roles in positive regulation of interleukin-6 and NF-KappB signaling; and MANBA participates in protein modification processes, specifically glycosylation.

However our study also identified two new proteins (TBCE and TPP2) implicated in T2D. TBCE was associated with a cis-pQTL (with chr1:235423298:C:T (negLog10p-value = 12.2), PWAS p-value = 1.46×10^− 5^, and MR FDR = 7.35×10^− 5^ on EUR-T2D). This locus passed genome-wide significance in the T2D-GWAS (Figure S11A), but no candidate genes within the locus were identified^5,6^. TBCE was reported to be part of the causal pathway underlying progressive encephalopathy with distal spinal muscular atrophy^33^. The other novel protein TPP2 had a trans-pQTL (chr8:9172650:G:A negLog10p-value = 10.6, PWAS p-value = 4.09×10^− 6^, and MR FDR = 4.23×10^− 4^ on EUR-T2D). This variant, however, was reported to be within a T2D risk locus passing the genome-wide significance threshold (Figure S11C). The nominated functional genes within this region were *ERT1* and *MFHAS1* by the TWAS and colocalization results.. TPP2 functions in intracellular amino acid homeostasis^32^.

Just like the proteomic results, there were no metabolites in common between EUR and AFR-prioritized results based in the triple analyses (MWAS, colocalization, MR). The only AFR-significant metabolite, the GlcNAc sulfate conjugate of C21H34O2 steroid**, had not been reported to be implicated with T2D before (Fig. 4E, Table S37). This metabolite was regulated by the genetic variant near *UGT3A1* (mQTL with chr5:35965868:A:C (negLog10p-value = 10.6), MWAS p-value = 7.79×10^− 4^, and MR FDR = 9.11×10^− 4^ on AFR-T2D) and it belonged to the partially characterized molecules. Interestingly, a similar metabolite (HMDB0001449) has been reported to be reduced in the blood of human patients with major depression^34^.

Of the four EUR metabolites associated with EUR T2D^5^ (Fig. 4E, Table S38), N-acetyl-isoputreanine was found to be associated with genetic variants in the *AOC1* gene. This gene has been nominated as a functional gene in two multi-ancestry GWA studies.^5,6^ However, this is the study linking variants in the *AOC1* gene with N-acetyl-isoputreanine as part of the causal pathway for T2D.^35^ Notably, this metabolite was the only one with a negative association in the EUR-specific T2D analyses. The other three metabolites implicated in T2D from our EUR-stratified analyses: 1-stearoyl-2-linoleoyl-GPE (18:0/18:2)*, 1-oleoyl-2-linoleoyl-GPE (18:1/18:2)*, 1-stearoyl-2-dihomo-linolenoyl-GPE (18:0/20:3n3 or 6)*, have not been reported as effector analyte in previous multi-ancestry T2D GWAS^5,6^. However, it is worth mentioning that 1-stearoyl-2-linoleoyl-GPE (18:0/18:2)* has been reported as significantly different between T2D cases and healthy controls by Zaghlool et al.,^31^. Our study serves as an example of integrating both genomics and metabolomics to support that this metabolite is part of the T2D pathway. Phosphatidylethanolamine (PE) metabolites, in general, have been implicated in T2D, non-alcoholic fatty liver disease, Parkinson’s disease, and Alzheimer Disease, based on previous metabolomic studies curated by HMDB.

To extend the XWAS and colocalization findings using an advanced statistical approach, we applied the recently published INTACT framework^36^ (Table S39–42). In summary, we found ancestry-matched T2D associations with six EUR and two AFR proteins, six EUR and one AFR metabolite (details in **Supplementary Notes**). These associations contained all the previously mentioned pairs, along with additional pairs related to one EUR protein (MANS4) and one AFR protein (BGAT), as well as two EUR metabolites (5-oxoproline and betaine). Notably, both proteins were reported as effector genes in previous multi-ancestry T2D GWAS^5,6^, whereas the two metabolites were not previously identified as such.

Moreover, to detect the patterns of the proteins and metabolites implicated here in T2D, we performed a cell-type-specific analysis using stratified-LDSC^37^. Regardless of ancestry, these proteins and metabolites displayed high expression in T cells and myeloid cells (Figure S13). Finally, to nominate druggable targets for repositioning, we queried the proteins and metabolites against the Drugbank database^38^. As a result, we found one EUR protein, Dtk, could be targeted by Fostamatinib, an FDA-approved drug used to treat chronic immune thrombocytopenia. On the other hand, the AFR protein, BGAT, exhibited targetability by 13 drugs, though none of them had FDA approval at the time of this study. Furthermore, two EUR metabolites, 5-oxoproline and betaine, could be targeted by pidolic acid and N,N,N-trimethylglycinium, respectively. Notably, pidolic acid has already been approved by the FDA to treat a family history of diabetes.

## Discussion

Our study involved large-scale multi-ancestral multi-omic plasma-based QTL mapping from the same cohort. Using XWAS, colocalization, and Mendelian randomization approaches, we identified key proteins and metabolites implicated in T2D. More importantly, our findings revealed ancestry-specific results. It is known that different ancestries have different genetic architectures of the same traits, here we extend the genetic findings to the downstream functional analytes underlying T2D. Specifically, our EUR-specific analyses uncovered five proteins (including three novel) and four metabolites (including three not previously reported) associated with T2D. Similarly, the AFR-specific analyses identified one previously unreported protein and one previously unreported metabolite.

Even we identified AFR-specific proteins and metabolites, the power of identifying unique signals in this ancestry is still lower than in EUR, as the sample size in the disease GWAS and omic data is much lower in AFR, leading to fewer disease-associated loci and xQTLs. Therefore, future research with larger disease GWAS and QTL maps from diverse populations is still necessary. In this study, we only nominate proteins or metabolites that met the stringent criteria of being identified through all three integrative strategies: XWAS, colocalization, and MR. This assures the highest confidence in these identified effectors being implicated in T2D. However, some proteins and metabolites survived in only two of the three criteria, which could warrant broader investigation.

There are four notable strengths of this study. First, it represents a large-scale research endeavor that encompasses multi-ancestry and multi-omics analyses, allowing for a comprehensive exploration of diverse populations and molecular trait layers at the same time. Second, we included trans QTLs into the conventional cis QTL framework^13,14^, enabling the discovery of more heritable features and expanding our understanding of the genetic underpinnings of the studied traits. In addition, we integrate this QTL data with FUSION, colocalization and Mendelian Randomization approaches to identify high confidence proteins and metabolites implicated on T2D. Our analyses provide additional support for some of the nominated effector genes (Dtk, C1QT4, and MANBA), but also nominated two new proteins in the EUR-specific analyses. One protein, TBCE, is located in a known locus, and we are nominated this protein as functional in this locus. As mentioned, in this study we not-only performed *cis*-QTL mapping but extended to *trans*-QTL as this can help to identify novel molecular interactions that otherwise will not be identified. The other novel protein identified in this study to be implicated on T2D is TPP2. The association of this protein with T2D, is through a *trans*-QTL with a SNPs located on a locus that includes *ERT1* or *MFHAS1* as potential effector genes. Additional studies are needed to determine how TPP2 and *ERT1* or *MFHSA1* interacts with each other, but this study provides strong evidence that these genes are part of the same pathway and it is a good example of how this unbiased analyses will identify new protein-protein interactions. Third, by performing ancestry-matched omics-disease integration, we enhanced the accuracy of our findings in comparison to previous studies that included ancestry-mixed data^5,6^. This approach may contribute to more precise identification of T2D effector genes within specific ancestral populations. Fourth, to ensure the robustness of our conclusions, we cross-referenced our findings against the two largest multi-ancestry T2D studies^5,6^. This stringent evaluation allowed us to determine whether our identified effectors had been reported previously or not, further reinforcing the significance of our results.

On the flip side, there are several limitations to consider in our study. First, our study did not integrate proteomics and metabolomics data due to the lack of colocalization between protein, metabolite, and the ancestry-matched T2D risk loci (data not show). Nonetheless, we believe that genetic colocalization of proteomics and metabolomics could exist if we were not solely focused on T2D-associated loci. Second, there were unequal sample sizes between participants of EUR and AFR ancestry. This discrepancy in sample sizes affected the power to detect QTLs, even though we were well-powered to identify the sentinel variants (Table S9). To mitigate this bias in identifying ancestry-specific findings, we employed standardized z-values that accounted for both effect size and standard error, rather than relying solely on p-values, when comparing the two ancestral groups. Third, our study utilized plasma bulk-tissue, which may not reflect the cell type of interest when studying T2D, such as pancreatic islets or beta cells. But as plasma circulates throughout the body^39^, our study holds value in investigating human metabolic disorders in general. Fourth, our study utilized one certain platform for measuring proteomics and the other for metabolomics, this can lead to platform-biased results. We, however, addressed this concern by querying our findings with external pQTL and mQTL studies that used both the same and different platforms. Fifth, our cohort consisted of participants with various disease statuses, including Alzheimer disease, frontotemporal dementia, and healthy individuals. We and other researchers, however, have reported that few pQTLs^21,40–42^ or mQTLs^25,43,44^ were status-specific. Thus, it suggests that the disease status is unlikely to have a significant impact, although further studies will be necessary. Sixth, we defined our participants based on genetic ancestry^45^, thus we used “African” ancestry rather than African American. We acknowledge our study may contain admixed participants, which could potentially underestimate the ancestry-specific features observed.

While our study focused on applying these plasma xQTLs to the study of T2D, it is worth noting that these QTL maps can be expanded to explore other diseases as well. These nominated proteins and metabolites are key intermediate phenotypes that can connect the genotype to the disease endpoint. Therefore, identifying these effectors in diverse populations may be an initial step toward developing more precise prediction models and therapies.

## Methods

### Ethics declarations

This project was approved by the ethics committee of the Washington University School of Medicine in St. Louis.

### Cohort information

All participants were recruited at the Knight Alzheimer Disease Research Center (Knight ADRC). In total, 3,170 participants from all genetic ancestries were selected for both proteomics and metabolomics profiling.

We used the TOPMed recommendations^46^ when defining ancestries based on genetic information (See the following section “Genotype QC, imputation, and population stratification”). Therefore, we used the terms of “European (EUR)” and “African (AFR)” when referring to participants recruited at the Knight-ADRC (USA) with European and African genetic backgrounds, respectively and regardless of the country of origin. Most participants recruited at the Knight-ADRC could be also classified as “European American” or “African American” in terms of race and ethnicity.

The plasma proteomic and metabolomisc datasets were generated from participants, which included 1,254 AD patients, 1,720 healthy controls, 34 frontotemporal dementia patients, and 162 individuals with an unclassified neurodegenerative disease. The cohort was a subset of the participants recruited from the Knight ADRC, which includes community-dwelling adults older than 27 years old via prospective studies of memory and aging since 1979. All participants recruited from the Knight ADRC are required to participate in core study procedures, including annual longitudinal clinical assessments, neuropsychological testing, neuroimaging, and biofluid biomarker studies. The corresponding genotype was a priori to choosing participants for profiling proteomics and metabolomics specifically in this study. Plasma samples were collected in the morning after an overnight fast, immediately centrifuged, and stored at −80°C.

### Proteomics data QC

In brief, 3,132 participants and 6,907 aptamers passed proteomics QC. 7,548 aptamers were measured before proteomics QC using the SOMAscan 7k platform^18^. Plasma proteomics data from all genetic ancestries were QCed with seven steps (details see **Supplementary Notes**): Step 1) Limit of detection, scale factor difference, and coefficient of variation; Step 2) IQR-based outlier expression level detections; Step 3) Remove Analytes and Samples with < 65% call rate (Figure S1A-B); Step 4) Re-calculate call rate for analytes and remove analytes with call rate < 85%; Step 5) Re-calculate missing rate for subjects and remove subjects with < 85% call rate cut-off (Figure S1C-D); Step 6) Back transformation into raw values; Step 7) Removal of Non-Human and analytes without protein targets and Final matrix.

### Metabolomics data QC

Briefly, 3,169 participants and 1,508 metabolites passed metabolomics QC. 1,718 metabolites were measured before QC with Metabolon HD4 platform^19^. Plasma metabolomics data from all genetic ancestries were QCed with 11 steps (details see **Supplementary Notes**): Step 1) Volume Normalization; Step 2) Sample Missingness; Step 3) Metabolite Missingness; Step 4) Fischer’s Exact Test and Differential Expression Check; Step 5) Minimum Value Imputation; Step 6) Remove non-informative metabolites; Step 7) IQR-based outlier detection; Step 8) Metabolites with < 50 data points; Step 9) Sample Outlier removal based on PCA; Step 10) Batch effects of metabolomics data; Step 11) Final Matrix and Back transformation of metabolite levels. Notably, for step 5, missing values were imputed. However, as mentioned earlier Xenobiotics are indeed expected to be missing, and imputing their values could skew the results. So, imputation is performed only for the non-xenobiotic group of metabolites.

### Genotype QC, imputation, and population stratification

At the pre-imputation stage (see **Supplementary Notes** for the details), the directly genotyped variants were kept agreeing to three criteria: (1) genotyping successful rate ≥ 98% per variant or per individual; (2) MAF ≥ 0.01; and (3) Hardy–Weinberg equilibrium (HWE) (P ≥ 1 × 10^− 6^). Imputation was performed on the TOPMed^3^ imputation server using the hg38 Version R2 reference panel. The TOPMed Imputation Reference panel contains information from 97,256 deeply sequenced human genomes. Imputed genotypes with imputation quality of R^2^ ≥ 0.3 were kept. At the post-imputation stage, the genotyped and imputed variants remained on the two criteria: (1) genotyping missing rate < = 90% per variant; (2) MAF > = 0.0005. Multiple genotyping arrays were included for this cohort. Including CoreEx, GSA_v1, GSA_v2, GSA_v3, Human1M.Duov3, NeuroX2, OmniEx, quad660 (Table S1). For each genotype array, we performed pre-imputation, imputation, and post-imputation separately. We merged all into one dataset before performing the QTL analyses (see **Supplementary Notes** for the details). Thus, the final number of genetic variants located on autosomal chromosomes was 10,448,203. In total, 3,081 out of 3,170 participants had corresponding genotype data.

Population stratification was performed using Plink1.9^47^ pca function, 2,598 participants were classified as EUR and 433 as AFR per principal component analysis (PCA) with the reference by 1000 genome project (Figure S2A-D, Table S2). We defined the genetic ancestry per genotype PCA anchored with participants from 1000 Genome Project within the black window defined per mean +/− 3 times of the standard deviation of each PC. Though all individuals in our grouping framework were within 3 standard deviations from the AFR participants in 1000 Genome project, we still cannot rule out the admixed populations from these participants. Relatedness was performed using plink1.9^47^ genome function on the IBD. Unrelated participants were defined as PI_HAT < 0.25. 2,395 EUR and 418 AFR participants were kept as unrelated participants.

### Identification of pQTLs

A linear regression model was used from plink2^47^ glm function for each protein. Protein-abundances were log-10 transformed first and z-scale normalized next. Covariates were age, sex, genotyping array types, genotype PC 1–10, and proteomics PC 1–2 (to correct such batch effects from the proteomics data alone: we identified two different batches when visualizing the scatterplots of proteomic PC1 and PC2 (Figure S1E). But after adjusting for the proteomic PC1 and PC2, the batch effect was corrected (Figure S1F)). Genotype array types included Quad660, CoreEx, GSA_v1, GSA_v2, GSA_v3, NeuroX2, Human1M.Duov3, when using as covariates, dummy variable included n-1 rather n. The final sample size for EUR and AFR pQTL analyses were 2,338 and 414 (Figure S3A, Table S3). The final numbers of proteins for EUR and AFR pQTL analyses were both 6,907 (Figure S3B, Table S4–5).

For cis and trans definitions, we used a window of the variants within 1 Mb upstream and downstream of the gene start site by which each protein was coded. P values for each variant–protein pair were estimated using an additive linear regression model. The cis threshold was 5×10^− 8^. For the trans-pQTL analysis, the number of PCs of EUR proteomics and AFR proteomics used as denominators were 1472 and 336, respectively. (The number of PCs was derived as the minimum PC number that cumulatively explains 95% of the variance for the proteomics expression matrix of each ancestry after QC.) Thus, the P-value threshold for EUR was 3.40×10^− 11^ (5×10^− 8^/1,472) and for AFR was 1.49×10^− 10^ (5×10^− 8^/336).

### Identification of mQTLs

A linear regression model was used from plink2^47^ glm function for each metabolite. Metabolite levels are first normalized by the median value given the same metabolite and log-10 is transformed next. Covariates are age, sex, genotyping array types, genotype PC1–10, and metabolomics PC 1–2. Genotype array types included Quad660, CoreEx, GSA_v1, GSA_v2, GSA_v3, NeuroX2, Human1M.Duov3, when using as covariates, dummy variable included n-1 rather n. The final sample size for EUR and AFR mQTL analyses was 2,392 and 417 (Figure S3A, Table S3). The final numbers of metabolites for EUR and AFR mQTL analyses were 1,483 and 1,413 (Figure S3B, Table S6–7). P values for each variant–metabolite pair were estimated using an additive linear regression model.

For the mQTL analysis, the number of PCs of EUR and AFR metabolomics used as denominators were 766 and 281, respectively. (The number of PCs was derived as the minimum PC number that cumulatively explains 95% of the variance for the metabolomics expression matrix of each ancestry after QC.) Thus, the P-value threshold for EUR was 6.53×10^− 11^ (5×10^− 8^/766) and for AFR was 1.78×10^− 10^ (5×10^− 8^/281).

### Filtering the inflation features

For the inflated features (i.e., associated with variants over 5/3/7/3 different chromosomes corresponding to EUR pQTL/AFR pQTL/EUR mQTL/AFR mQTL [the thresholds are collected empirically]), we first removed the variants given this feature with MAF < 0.05 and genotyping call rate < 97%. If we found the features were still inflated, we removed the features eventually. The unique features of removal were listed below: EUR proteomics: 142 aptamers; AFR proteomics: 132 aptamers; EUR metabolomics: six metabolites; AFR metabolomics: five metabolites.

### Annotation of the xQTLs

To annotate our QTL findings, we used the command line tool Variant Effect Predictor (VEP^48^) from the Ensembl-version107. We used the default options for all four QTL maps.

### Replication of xQTLs with the external studies

To replicate our QTL findings, we queried all study-wide feature-variant pairs from our study against several largest external studies. These studies all released their full summary statistics and set the genetic coordinates in the hg38. The proxy variant was defined as LD r^2 > = 0.8 using the reference at TOPMed^3^ WGS data curated by the tool TOP-LD^49^.

To replicate our proteomics findings, we used four datasets from three studies. Ferkingstad et al.,^23^ used 35k participants of European ancestry and the SOMAscan 5k platform to measure plasma proteome. Surapaneni et al.,^20^ used 466 participants of African ancestry and the SOMAscan 7k platform to measure serum proteome. Sun et al.,^15^ used 34k participants of European ancestry as well as 931 participants of African ancestry and the OLINK 3k platform to measure plasma proteome. We set six categories when comparing the study-wide significant findings from this study and its corresponding external studies: 1) validated with a p-value below the Bonferroni-corrected study-wide threshold (5×10^− 11^) account for 1000 features; 2) known with a p-value below the genome-wide threshold (5×10^− 8^); 3) replicated with a p-value below the nominal threshold (5×10^− 2^); 4) not replicated with a p-value greater or equal to the nominal threshold; 5) not reported with a matching protein but a missing proxy variant; 6) not reported with a non-matching protein.

To replicate our metabolomics findings, we used three studies. Yin et al.,^25^ used 6,136 participants of European ancestry from Finland and the Metabolon HD4 platform to measure plasma metabolome. Chen et al.,^26^ used 8,299 participants of European ancestry and the Metabolon HD4 platform to measure plasma metabolome. Rhee et al.,^27^ used 687 participants of African ancestry and the Broad Institute platform to measure plasma metabolome. For mQTLs of African ancestry by Rhee et al. 2022, the number of metabolites overlapping between their platform (Broad Institute) with our platform (Metabolon HD4) was 207. This overlap was performed via HMDB-ID matching, rather than chemical name (Table S8). We set four categories when comparing the study-wide significant findings from this study and its corresponding external studies: 1) validated with a p-value below the Bonferroni-corrected study-wide threshold (5×10^− 11^) account for 1000 features; 2) known with a p-value below the genome-wide threshold (5×10^− 8^); 3) replicated with a p-value below the nominal threshold (5×10^− 2^); 4) not replicated with a p-value greater or equal to the nominal threshold; 5) not reported with a matching metabolite but a missing proxy variant; 6) not reported with a non-matching metabolite.

### Definition of LD block and pleiotropy

To define LD blocks, we used the 1000G EUR (1703 blocks) as the reference population per ldetect by Berisa and Pickrell^22^. We performed liftover to map the hg19 coordinates into hg38. We next used the index to group the variants and obtained the pleiotropic region, which was the index associated with multiple molecular traits. For proteomics, we used karyoploteR^50^ package to visualize the top findings as an ideogram. For metabolomics, we used circlize^51^ package to visualize the top findings as a chord diagram.

### Identification of ancestry-specific QTLs

Ancestry-specificity was defined as fold-change over 10-fold or below 0.1-fold between the Z-normalized effect sizes (beta divided by standard error) of the protein-variant pairs or metabolite-variant pairs given the same variants. The fold-changes of the same feature-variant pairs were also calculated after setting 3 bins of MAF as 0 to 0.01, 0.01 to 0.05, and 0.05 to 0.5.

MASH method^29^ (implemented as mashR package) was also used. Briefly, after fitting the model into the mash function with the beta and standard error of the same QTLs from both EUR and AFR datasets as the input plus the covariance matrices set up given the same input. The fold-change of posterior means of the protein-variant pairs or metabolite-variant pairs given the same variants were calculated. The same thresholds of 10-fold and 0.1-fold were used to determine QTL sharing or not.

Boxplots were drawn with the ggplot2 package; Locus-zoom plots were drawn with the LocusZoom.js tool^52^.

### Power analyses of ancestry-specific QTLs

We performed two separate power analyses using the powerEQTL.ANOVA function from the R package powerEQTL^53^ is listed below:

- We calculated the power values for all our current sentinel variants from each of the four QTL sets after splitting them into ancestry-shared and ancestry-specific subtypes.
  Given the input of MAF, the average standardized effect size per ancestry-specificity, the sample size, and using the genome-wide significance thresholds (FWER = 0.05, nTests = 1e6), we found all variant-feature pairs with a power of 0.8 or more (Table S9).
- We next fixed the effect size and number of tests, while varying MAF per each of the four QTL sets. We split the MAF into minimum, average, and maximum by ancestry-shared and specific xQTLs. We empirically learned the MAF and the average standardized effect size and used the genome-wide significance thresholds for consistency between ancestries and specificity of xQTLs.
  We found that min-MAF led to underpowered findings, especially in the ancestry-specific xQTLs. For example, in the EUR mQTL set, the power was 0.007 for identifying EUR-specific findings in AFR given minMAF from ancestry-specific variants (Table S10). On the other hand, the power turned to 1 for identifying the EUR-shared mQTL in AFR given minMAF from shared variants.

### Cross-reference of the ancestry-specific xQTLs with external studies

We cross-referred to Zhang et al.,^13^ for proteomics to examine the proportion of the ancestry-specific pQTLs. We calculated the percentage of ancestry-specific pQTLs using the variable “EA-specific” as TRUE over all EUR-pQTLs in their Supplementary Table 3.1 and the variable “AA-specific” as TRUE over all AFR-pQTLs in their Supplementary Table 3.2.

We cross-referred Rhee et al.,^27^ for metabolomics datasets to check the proportion of the ancestry-specific mQTLs. We derived the percentage of ancestry-specific mQTLs with ones outside the 10-fold-change per the Z-normalized effect sizes (beta divided by standard error) of EUR and AFR over the total 45 mQTLs in their Table 2.

### T2D risk GWAS

We used the two population-scale T2D risk GWAS from EUR and AFR, separately.

For EUR T2D risk GWAS, we used the summary statistics from the study by Mahajan et al.,^5^ covering 80,154 cases and 853,816 controls. The full summary statistics for EUR and multi-ancestry GWAS were available at http://diagram-consortium.org/downloads.html; while AFR GWAS was not available as of September 2023.

Thus, for AFR T2D risk, we turned to another study from Vujkovic et al.,^6^ containing 24,646 cases and 31,446 controls. The partially significant (p < 1×10^− 4^) summary statistics for AFR GWAS were available at https://www.ncbi.nlm.nih.gov/gap/advanced_search/?TERM=pha004944.1. To perform the downstream QTL and disease integration, we aggregated the non-overlapping and non-partially significant (p > = 1e-4) variants from the multi-ancestry GWAS by Mahajan et al.,^5^ to the original AFR GWAS as the final full summary statistics for the AFR.

### PWAS weight calculation

A modified version of FUSION^12^ was used. The SNP-based heritability of each protein SOMAmer was estimated using the GCTA GREML^54^ tool. The proteins with negative h^2^ values were removed before performing the weight calculation. The window size of the sentinel QTL region was± 1 Mb. For the proteins associated with more than one genetic region, all variants from each region were included in the weight calculation. Using the FUSION R package, we constructed imputation models for 881 AFR and 2,400 EUR SOMAmers. The imputation model for a SOMAmer was trained by the best models (out of Elastic Net, TOP1, and BLUP) using all variants in ±1 Mb upstream and downstream of the sentinel pQTL sites of the target protein. The Elastic Net model was refitted using all data and the tuning parameters per 5-fold cross-validation.

### PWAS association test with T2D

A modified version of FUSION^12^ was used. We used the 881 (AFR) and 2,400 (EUR) imputation models to perform the PWAS on the ancestry-matched T2D risk. The tool Functionally-informed Z-score Imputation (FIZI^55^) was used first to impute the summary statistics of AFR T2D risk (Vujkovic et al., 2020^6^) and EUR T2D risk (Mahajan et al., 2022^5^) with the in-sample reference linkage-disequilibrium (LD) information.

The multiple testing corrections for the PWAS results were selected per the total number of imputation models for all weight-non-missing plasma proteins (P value < 0.05/797 in AFR and 0.05/2,285 in EUR). The Z value from PWAS was used to determine the effect size of protein-T2D associations within each ancestry.

### MWAS weight calculation

Similar to the above PWAS weight calculation section, a modified version of FUSION^12^ was used. The SNP-based heritability of each metabolite was estimated using the GCTA GREML^54^ tool. The metabolites with negative h^2^ values were removed before performing the weight calculation. The window size of the sentinel QTL region was ± 1 Mb. For the same metabolites associated with more than one genetic region, all variants from each region were included in the weight calculation. Using the FUSION R package, we constructed imputation models for 60 AFR and 403 EUR metabolites. The imputation model for a metabolite was trained by the best models (out of Elastic Net, TOP1, and BLUP) using all variants in ± 1 Mb upstream and downstream of the sentinel mQTL sites of the corresponding metabolite. The Elastic Net model was refitted using all data and the tuning parameters per 5-fold cross-validation.

### MWAS association test with T2D

Similar to the above PWAS association test section, a modified version of FUSION^12^ was used. We used the 60 (AFR) and 403 (EUR) imputation models to perform the MWAS on the ancestry-matched T2D risk (AFR T2D risk (Vujkovic et al., 2020^6^) and EUR T2D risk (Mahajan et al., 2022^5^)) after FIZI imputation.

The multiple testing corrections for the MWAS results were selected per the total number of imputation models for all weight-non-missing plasma proteins (P value < 0.05/401 in AFR and 0.05/58 in EUR). The Z value from MWAS was used to determine the effect size of metabolite-T2D associations within each ancestry.

### Identification of ancestry-specific XWAS-T2D findings

Comparison of the p-values with the same analyte-T2D associations given the same trait from the two ancestries. If the analyte-T2D association from each ancestry was significant, the analyte was ancestry-shared. If the analyte-T2D association from only one ancestry was significant, the analyte was ancestry-specific. The Miami plots for PWAS and MWAS comparing EUR and AFR findings were plotted using the R package hudson^56^.

### Colocalization of molecular traits (proteins/metabolites) and T2D

After PWAS and MWAS, only the significant proteins/metabolites were kept. To remove the LD bias in the significant PWAS/MWAS findings, we performed colocalization analysis using both coloc.abf function from R package coloc v3.1^7^ and coloc.susie function from R package coloc v5.1^8^ with a wrapper for susie_rss function from susieR^57,58^ package. We next set the window size to +/− 1Mb centering on IV per trait-T2D pair. We used the default priors, with p1 as 1×10^− 4^, p2 as 1×10^− 4^, and p12 as 1×10^− 5^. Evidence for colocalization was assessed using the posterior probability (PP) for hypothesis 4 (indicating there is an association for both protein and disease and they are driven by the same causal variant(s)). We used PP.H4_final > 80% as a threshold to suggest that associations were highly colocalized. Under the assumption of only a single causal variant, we used the PP.H4 from coloc.abf output of the trait-disease pair. Under the assumption that multiple causal variants exist^57^, we used the maximum PP.H4 of multiple credible sets from coloc.susie output.

### Mendelian randomization of proteins or metabolites on T2D

After PWAS/MWAS, only the significant proteins or metabolites were kept. To further infer the causal effects of the proteins or metabolites on T2D risk, we performed MR analyses after removing pleiotropic QTLs. These pleiotropic QTLs were defined variants within the pleiotropic regions given a minimum feature: 6 proteins for EUR and AFR proteomics: and 11 metabolites for EUR and AFR metabolomics). After keeping the rest variants as the valid instrumental variables, we used R package TwoSampleMR^9^ v0.5.7, which includes two primary methods: For every single SNP, the most basic way, Wald ratio, was used; For multiple SNPs, inverse variance weighted (IVW) estimator was used. All pQTL or mQTLs used as instrumental variables have F-statistics greater than 10 and with study-wide significance after the in-sample LD clumping with plink1.9^47^. As not all features within each cohort are independent, we used a false discovery rate (FDR) < 0.05 as our multiple-test correction approach.

### Evidence integration of proteins/metabolites on T2D

After intersecting PWAS or MWAS and coloc, we consider MR as an extra layer of evidence. We first intersected features with XWAS passing Bonferroni correction; coloc passing PP.H4 > 0.8 and MR FDR < 0.05 as significant proteins/metabolites. We next used the INTACT framework^36^ to compute an updated PP with XWAS and coloc as input. Given the INTACT PP > 0.8, we further intersected with MR FDR < 0.05 as significant proteins/metabolites.

### Effector genes of proteins/metabolites on T2D

We first assembled the effector genes of T2D risk GWAS from two multi-ancestry studies^5,6^. For the study by Mahajan et al., we combined their missense annotations, colocalization with seven-tissue eQTL and plasma pQTL, pcHi-C annotations. In total, 834 unique genes were nominated by the authors. For the study by Vujkovic et al., we combined their missense annotations, TWAS, and colocalization results from 52 tissue eQTLs. In total, 754 unique genes were nominated by the authors. We next queried our protein and metabolite findings using the nearest gene to the genetic locus. If the gene can be found in the effector list, we define it as the finding that was reported.

### Cell-type specificity analysis of the proteins/metabolites on T2D

After intersecting INTACT and MR results, we performed cell-type specificity analysis of the proteins/metabolites on T2D using S-LDSC^37^ with the cell-type annotation from ImmGen. Overall, 295 cell subtypes were used and grouped into five major cell types: B, Myeloid, Natural Killer (NK), T, and other cells. The input for S-LDSC was each protein or metabolite genome-wide summary statistics. The output of the S-LDSC was the regression coefficient of the cell type-specific annotation from ImmGen as well as the p-value of the coefficient for each feature. We then ranked the feature by the p-value and used the top cell type as the enriched cell type for this feature. We used two input files: one from the INTACT and MR integration results; and the other from all features with at least one QTL. We calculated the fold-change of the two sets given the same feature-ancestry combination (i.e., EUR proteins, AFR proteins, EUR metabolites, and AFR metabolites).

### Druggable target query of the proteins/metabolites implicated in T2D

To nominate druggable targets for repositioning, we queried the proteins and metabolites against the drugbank database^38^ (drugbank_5.1.10.db) downloaded locally. For proteins, we first downloaded the csv file for all Drug Target Identifiers (https://go.drugbank.com/releases/latest#protein-identifiers). The uniprotID and drugbankID were linked in the csv file. We next used the proteins with an overlapping uniprotID to query the corresponding drugbankID via the drugbankR R package (https://github.com/girke-lab/drugbankR). For metabolites, we first used the hmdbID to query via the hmdbQuery R package (https://github.com/vjcitn/hmdbQuery) and extracted the “drugbank_id” for the final query via the drugbankR.

### PheWeb Browser for interactively visualizing QTL datasets

To assist users in navigating our QTL results, we implemented the PheWeb^28^ to visualize all 16710 traits (EUR-proteins: 6907; AFR-proteins: 6907; EUR-metabolites: 1483; AFR-metabolites: 1413). The URL is https://ontime.wustl.edu/, all aligned to the hg38 genomic coordinates.

## Data Availability

The datasets generated during this study are available in the BOX folders listed below: EUR-proteomics: https://wustl.app.box.com/folder/246498133407 AFR-proteomics: https://wustl.app.box.com/folder/246495587213 EUR-metabolomics: https://wustl.app.box.com/folder/246497314974 AFR- metabolomics: https://wustl.app.box.com/folder/246496814203 The PheWeb browser for visualizing all plasma omics QTL results is at https://ontime.wustl.edu/.
